# DeepPeptide predicts cleaved peptides in proteins using conditional random fields

**DOI:** 10.1093/bioinformatics/btad616

**Published:** 2023-10-09

**Authors:** Felix Teufel, Jan Christian Refsgaard, Christian Toft Madsen, Carsten Stahlhut, Mads Grønborg, Ole Winther, Dennis Madsen

**Affiliations:** Department of Biology, University of Copenhagen, Ole Maaløes Vej 5, Copenhagen 2200, Denmark; Digital Science & Innovation, Novo Nordisk A/S, Novo Nordisk Park, Måløv 2760, Denmark; Digital Science & Innovation, Novo Nordisk A/S, Novo Nordisk Park, Måløv 2760, Denmark; Global Translation, Novo Nordisk A/S, Novo Nordisk Park, Måløv 2760, Denmark; Digital Science & Innovation, Novo Nordisk A/S, Novo Nordisk Park, Måløv 2760, Denmark; Global Translation, Novo Nordisk A/S, Novo Nordisk Park, Måløv 2760, Denmark; Department of Biology, University of Copenhagen, Ole Maaløes Vej 5, Copenhagen 2200, Denmark; Department of Applied Mathematics and Computer Science, Technical University of Denmark, Lyngby 2800, Denmark; Digital Science & Innovation, Novo Nordisk A/S, Novo Nordisk Park, Måløv 2760, Denmark

## Abstract

**Motivation:**

Peptides are ubiquitous throughout life and involved in a wide range of biological processes, ranging from neural signaling in higher organisms to antimicrobial peptides in bacteria. Many peptides are generated post-translationally by cleavage of precursor proteins and can thus not be detected directly from genomics data, as the specificities of the responsible proteases are often not completely understood.

**Results:**

We present DeepPeptide, a deep learning model that predicts cleaved peptides directly from the amino acid sequence. DeepPeptide shows both improved precision and recall for peptide detection compared to previous methodology. We show that the model is capable of identifying peptides in underannotated proteomes.

**Availability and implementation:**

DeepPeptide is available online at ku.biolib.com/DeepPeptide.

## 1 Introduction

Peptides fulfill a wide range of functions throughout life, ranging from antimicrobial peptides for defense against competitor species in bacteria to endocrine signaling hormone peptides in higher organisms. Moreover, an increasing number of drugs are based on engineering naturally occurring peptides (Wang 2022). Given their significance, computational prediction of peptides and their properties has gained wide interest and has successfully been applied to the identification of novel natural active peptides (Foster *et al.* 2019, Ma 2022, Madsen 2022). However, most recent work has focused on classifying peptides that were first observed experimentally, precluding fully computational peptide discovery. In this work, we focus on detecting potential natural peptides directly from readily available reference proteome data. Building upon recent advancements in deep learning methods for protein sequence analysis, we introduce DeepPeptide for predicting cleaved peptides in precursor protein sequences.

Predicting peptides first and foremost requires defining the concept of a peptide. Generally, peptides are understood to be short amino acid (AA) sequences. However, further characterization becomes increasingly imprecise, with no clear upper bound that defines the border between what is to be considered a peptide or a short protein (Bodanszky 1988). Moreover, in a context-oriented more toward cell biology, the notion of peptides being produced from precursor proteins through protease cleavage—as it is typically the case for hormones—is often included, even though this biological mechanism does not imply a short length of the resulting product (Akbarian *et al.*, 2022). Also multichain proteins are generated by cleavage, and within the context of such a protein, a chain would typically not be considered a peptide, even if it was very short in length (Klein 2018). Additionally, the products of small open reading frames are often considered to be peptides due to their length, while there is no protease activity involved in their expression (Chen *et al.* 2022).

**Figure 1.**
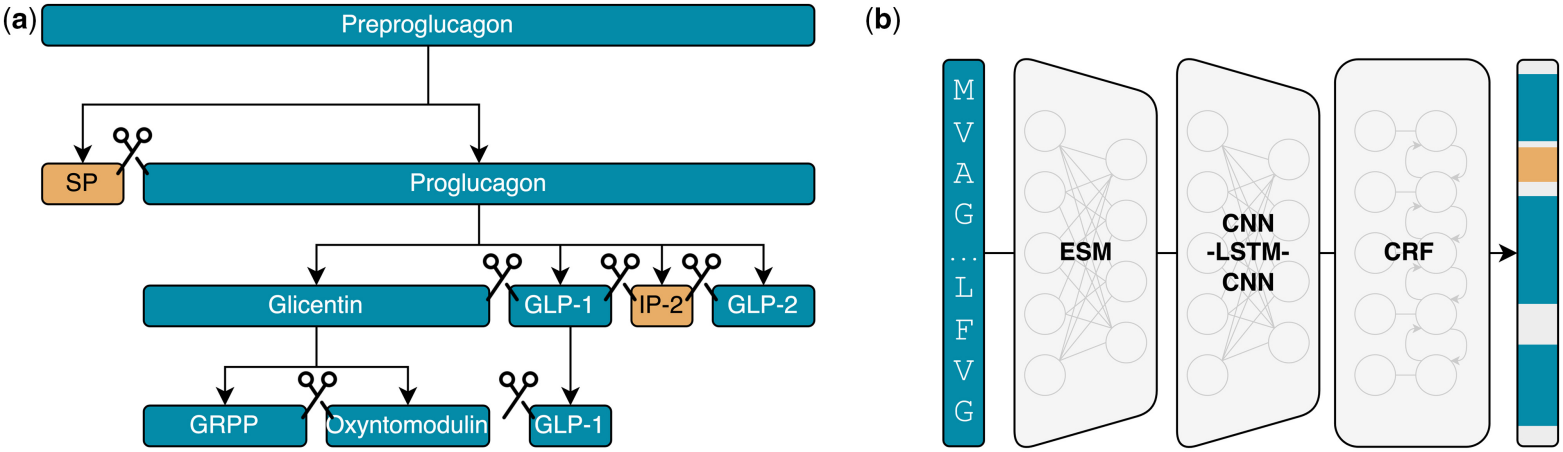
(a) Peptide synthesis *in vivo*. A precursor protein (Preproglucagon) is translated and processed through a cascade of proteases that iteratively cleave the backbone into smaller fragments, releasing peptides (SP, signal peptide; GLP-1, glucagon-like peptide 1; IP-2, intervening peptide 2; GLP-2, glucagon-like peptide 2; GRPP, glicentin-related polypeptide). The figure describes the processing in the human intestine. (b) The DeepPeptide model. A protein sequence is encoded by the ESM pLM and the resulting representation passed through a CNN-LSTM-CNN neural network. The neural network computes the emissions for a CRF layer that predicts the positions of peptide products within the sequence.

As evident from this brief discussion, there is no common consensus as to what precisely the term peptide entails. Within the context of DeepPeptide, we follow previous works on peptide modeling (Lamiable 2016) and settle for sequences in the range of 5–50 AAs. We define peptides as molecules that are produced by targeted proteolysis of precursor proteins, as exemplified by the well-known example Glucagon (Fig. 1a) (Sandoval and D’Alessio, 2015).

Previous work such as PeptideLocator (Mooney 2013) approached the problem of predicting peptides in precursor proteins as a position-wise classification problem, with the model predicting an individual probability of being observed in a peptide for each AA in the sequence. While this approach has proven capable of revealing some “peptide hotspots” within a protein sequence, it falls short of identifying the actual peptide sequences. We instead propose to treat peptide prediction as a sequence labeling problem using a conditional random field (CRF) (Lafferty *et al.*, 2001).

The CRF-based approach allows us to optimize a joint likelihood over the full sequence, instead of treating positions as independent. Furthermore, by decoding the CRF using the Viterbi algorithm (Viterbi 1967), this modeling framework allows us to predict discrete peptide borders directly without any post-processing of output probabilities (Fig. 1b). In addition to predicting peptide borders, we extend the task to also cover propeptides as defined by UniProt (The UniProt Consortium 2021). Both peptides and propeptides are endogenous cleaved peptide products of precursor proteins. In general, peptides are held to have a well-defined biological activity, whereas propeptides are without a likely independent function after cleavage.

CRFs model sequences as paths through a predefined state space. We design our state space with one state for not being in a peptide, and 50 states each for being in a propeptide or peptide, corresponding to positions 1–50 (Supplementary Fig. S1). We further constrain the transitions between the states to enforce predictions with a minimum length of 5 as well as preventing any spurious transitions from peptide to propeptide.

To predict the emission scores for the CRF, we train a CNN-LSTM-CNN neural network (Almagro Armenteros 2019) on top of precomputed ESM protein language model (pLM) embeddings (Rives *et al.* 2021, Lin *et al.* 2023). The combination of pretrained pLMs with CRFs has previously achieved state-of-the-art results on protein sequence annotation tasks (Hallgren 2022, Teufel 2022).

## 2 Materials and methods

### 2.1 Training data

For training and evaluation, we gathered all manually curated protein sequences annotated with peptides or propeptides from UniProt Release 2022_01, excluding viral proteins and protein fragments from the query. We discarded all proteins that are annotated with a peptide that covers the full-length range of the mature protein, as these are not peptides in the sense of being proteolytically released from a precursor protein. The same was done for all peptides that have full coverage together with an annotated signal or transit peptide. We removed sorting signals that are annotated as propeptides by PROSITE (Sigrist 2013) ProRules PRU00477 and PRU01070. We filtered the peptide annotations for a length range of 5–50 AAs and discarded all proteins that have no peptides within this range (Supplementary Fig. S2). For peptides, this length range covers 90% of the total annotations. The lengths of propeptides are more varied, with 63% falling in this range.

Naively, this data selection criterion encodes a prior that any protein sequence contains at least one peptide. However, we did not observe such spurious prediction behavior in the trained models when making predictions on whole proteomes (Table 3).

**Table 3.** DeepPeptide predictions on reference proteomes of model organisms compared to UniProt annotations for the same proteins.^a^

	UniProt		DeepPeptide	
Organism	Peptides	Propeptides	Peptides	Propeptides
*Homo sapiens*	352	394	458	815
*Mus musculus*	254	407	508	921
*Drosophila melanogaster*	126	83	355	1093
*Arabidopsis thaliana*	105	236	474	1346
*Saccharomyces cerevisiae*	56	99	101	501
*Pan troglodytes*	43	25	355	869
*Danio rerio*	14	31	205	1319
*Oryza sativa*	5	32	676	959
*Zea mays*	7	8	346	955

a Uniprot annotations that pass the DeepPeptide training data selection criteria were counted.

UniProt peptide annotations were used to generate per-position label sequences for each protein sequence. We generated our labels following the state space model that is capable of modeling peptides and propeptides of a variable length in the range of 5–50 AAs (Supplementary Fig. S1).

We applied GraphPart (Teufel *et al.* 2023) to homology—partition the protein sequences into 5-fold at maximum 30% Needleman–Wunsch pairwise identity between sequences in different folds. To keep fold sizes similar, we executed the tool using the –no-moving flag, as indicated in the documentation. After GraphPart partitioning, we obtained a dataset of 7623 protein sequences total (Table 1).

**Table 1.** Summary statistics of the DeepPeptide dataset.^a^

Group	Peptides	Propeptides	Proteins	Mean annotations per protein
Prokaryotes	104	523	531	1.18
Eukaryotes	6065	6836	7092	1.82
Thereof plants	227	711	742	1.26
Thereof chordata	3077	2790	3117	1.88
Thereof mammalia	1763	1697	1995	1.73
Total	6169	7359	7623	1.77

a UniProt taxonomy annotations were used. Plants refer to Viridiplantae.

To avoid a situation where different kinds of peptides are unevenly distributed between partitions, which could easily arise in the given dataset where the labeled classes are highly heterogeneous and unlikely to be generated by the same biological process, we performed clustering prior to partitioning. These cluster assignments were used for balancing the 5-fold so that artificial distribution shifts by random fold assignment are minimized. We based our clustering on the flanking motifs of the peptides, as these are presumably the main causal feature that gives rise to peptide cleavage. We extracted the two AAs before the N and after the C terminus of each peptide. To capture biochemical characteristics, we encoded each AA using ESM-2 (Lin *et al.* 2023) and concatenated the resulting four embedding vectors. On the concatenated vectors, we performed *k*-means clustering. We found that a value of *k* = 50 yields a useful number that captures meaningful motifs while still being amenable to partitioning (Supplementary Fig. S3). We note that this is not the strictly optimal number of clusters, which presumably lies higher. However, in the given dataset, the empirical optimum will yield singleton clusters that cannot be meaningfully used for partitioning sequences.

### 2.2 Model architecture

The peptide prediction task is formulated as a sequence-to-sequence mapping from the AA sequence *x* to the label sequence *y* with class labels “None,” “Peptide,” or “Propeptide” at each position. The model architecture consists of three sequence-to-sequence elements: a pretrained pLM encoder, a neural network for further feature extraction, and a CRF for structured prediction. We use the ESM-2 pLM with 650 million parameters to embed protein sequences, obtaining a hidden state embedding vector **h**_*t*_ of length 1280 for each position *t* in the sequence. These pLM embeddings serve as input features for a CNN-LSTM-CNN neural network that computes the emission score vectors ψ(*h*) for the CRF. This architecture has previously shown good performance for detecting protease cleavage sites within the context of signal peptides (Almagro Armenteros *et al.* 2019).

A linear-chain CRF is a state-space model that allows adjacent positions in the sequence to have dependent state labels and thereby make structured predictions. It does so by learning a transition matrix φ that encodes the unnormalized probability of transitioning between states at adjacent positions. From the emission scores ψ(*h*) and state transition parameters φ, one can compute the most likely state sequence with Viterbi decoding. We use the state space of the CRF to additionally encode the peptide length constraint of 550 AAs. We achieve this with the structured state-space model with 101 states in total as outlined in Supplementary Fig. S1.

In general, a CRF requires the computation of an emission score (logit) for each state at each position. However, as our state-space model serves the purpose of enforcing length constraints, rather than encoding 101 distinct biological classes, we do not compute an emission score for each state. Rather, we compute three emission scores for the classes {Peptide, Propeptide, None} and share the score between all states associated with the same class label.

The log likelihood can be computed using the forward algorithm and the parameters of the feature extraction neural network and the parameters for the allowed transitions are learned end to end.

### 2.3 Training and inference

The model is trained for 50 epochs with early stopping. We use the mean *F*1 score of propeptides and peptides as the stopping metric. Hyperparameters are optimized within the inner loop of a 5-fold nested cross-validation using Optuna (Akiba *et al.* 2019) (Supplementary Tables S1 and S2). For proteins with overlapping peptide annotations, we randomly sample one nonoverlapping annotation in each iteration and use it for generating the label sequence. During training, we keep the weights of the pLM frozen and only optimize the prediction model. As loss function, we minimize the negative log-likelihood of the CRF, where *Z*(*h*) is the normalization constant of the distribution *P*(*y*|*h*):


(1) $$-\log(P\left(y|h\right))=-\log(\log\left(Z\left(h\right)\right)-\log(\exp(\sum_{t=1}^{T}\psi \left(h_{t}\right)+\varphi (y_{t},y_{t-1})))$$

We use the Viterbi algorithm to predict the most likely label sequence. Additionally, for visual interpretation of predictions, we compute the marginal probabilities using the forward-backward algorithm (Supplementary Note S1). For both outputs, we simplify the displayed results by merging the 50 states of the peptide and propeptide branch into a single state each.

When applying the model to new data, we ensemble the 5 × 4 predictors trained in nested cross-validation. All 20 neural network models are run to compute emissions, these are averaged and passed to a CRF layer whose weights are the averages of the weights of the 20 CRF models. Due to each CRF weight having a predefined interpretable role, this simple averaging is possible, as opposed to general neural network models. The averaged CRF computes the most likely path. The marginal probabilities are computed by taking the mean over 20 CRF model forward-backward algorithm outputs (Supplementary Fig. S4).

### 2.4 Evaluation

For computing peptide-level metrics, we convert the output labels of each protein into a list of (start, end) tuples describing the positions of the predicted peptides. This list of predicted positions is then compared to the start and end positions of true peptides. For true positives (TP), we require that both the start and end position of a predicted peptide match the start and end position of a true peptide within a given tolerance threshold around the true positions. Remaining predicted peptides are false positives (FP), and true peptides with no match are false negatives (FN). True negatives cannot be defined in a meaningful way and are not required for the calculation of precision and recall. If the ground truth annotations contain overlapping peptides, we treat the whole cluster of overlapping peptides as a single peptide for scoring. It counts as correctly predicted if the model predicts any of the overlapping peptides correctly. From precision and recall, we compute the *F*1 score which we use for best checkpoint selection during training.


(2) $$\mathrm{Precision}=\;\frac{\mathrm{TP}}{\mathrm{TP}+\mathrm{FP}}$$

(3) $$\mathrm{Recall}=\;\frac{\mathrm{TP}}{\mathrm{TP}+\mathrm{FN}}$$

(4) $$F1=\;\frac{2\;*\;\mathrm{Precision}*\mathrm{Recall}}{\mathrm{Precision}+\mathrm{Recall}}$$

To compute the same metrics for PeptideLocator, we threshold its predicted probabilities at the default threshold of 0.5. From the resulting binary label sequences, we extract all contiguous positive segments. We consider these positive segments as predicted peptides and collect their start and end positions, from which we proceed as outlined earlier. Experiments confirmed that the default is a sensible choice (Supplementary Fig. S5). We only use the peptide part of the data and ignore propeptides when evaluating PeptideLocator.

In nested cross-validation, we compute precision and recall for each of the 20 models on their respective test set and report the means and standard deviations. To generate a confusion matrix, this approach would be impractical, as it yields 20 confusion matrices that would need to be aggregated. Instead, we combine the test set predictions from all 20 models and compute the confusion matrix from the combined data. Note that this implies that each data point contributes four counts to the confusion matrix, as it has four independent predictions.

## 3 Results and discussion

We evaluate model performance at a 3-AA tolerance window around the true start and end positions of peptides to account for uncertainty and ambiguity in the peptide annotations (Orskov 1989).

**Figure 2.**
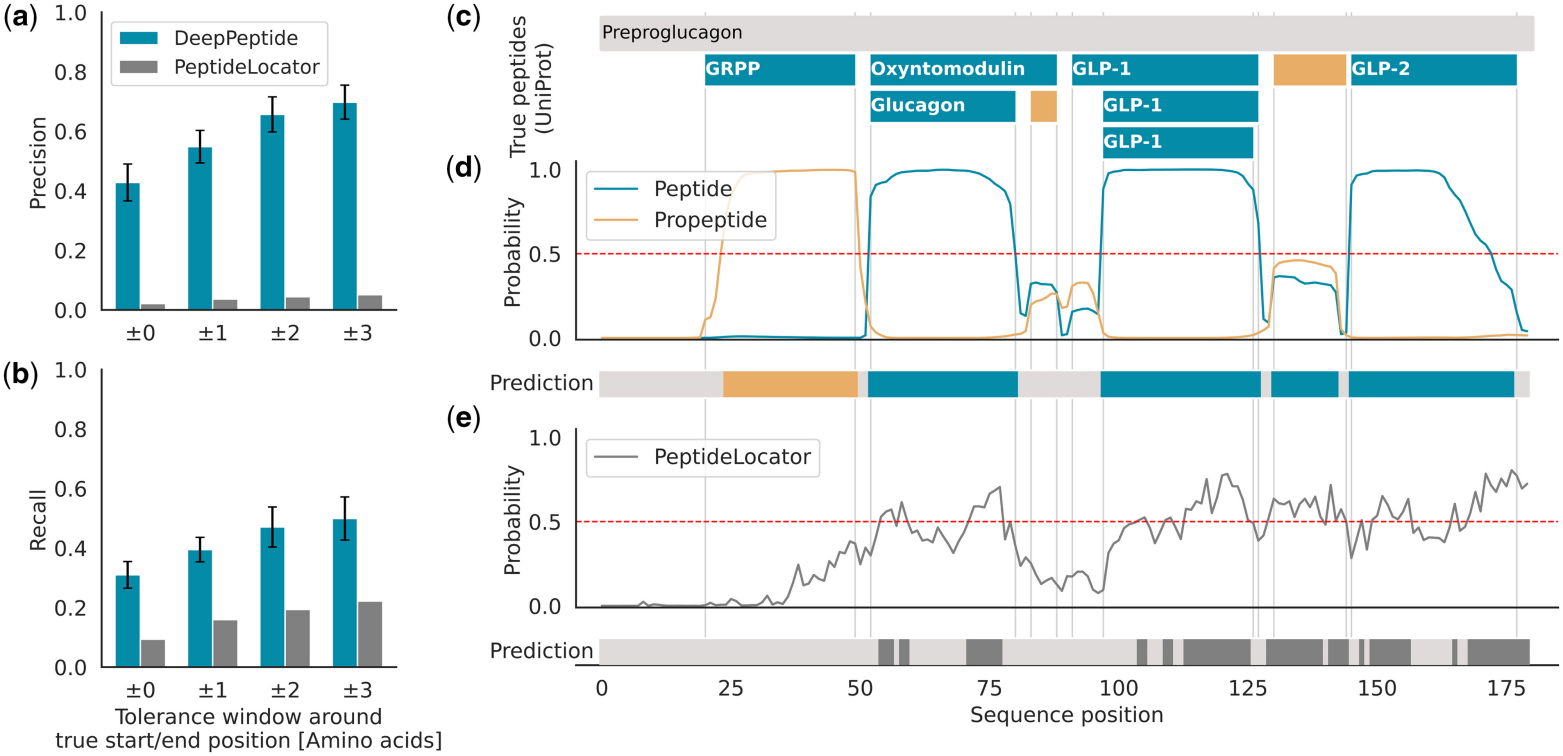
(a, b) Precision and recall of DeepPeptide and PeptideLocator at different tolerance thresholds. Metrics were computed in nested cross-validation, reporting the average and standard deviations over 20 models on their held-out test fold. (c) Annotated peptides and propeptides in Uniprot. (GRPP, glicentin-related polypeptide; GLP-1, glucagon-related peptide 1; GLP-2, glucagon-related peptide 2. The propeptides are not named). (d) Example model output of DeepPeptide for the protein sequence of human Preproglucagon (Uniprot ID P01275). The lines represent the predicted marginal probabilities, the colored bar indicates predicted peptides and propeptides in the top scoring path. The prediction was taken from the cross-validated model that trained on folds 0, 2, 4, validated on 3, and tested on 1. (e) Prediction of PeptideLocator for the same sequence.

We find that DeepPeptide reaches a precision of 0.68 and a recall of 0.49 at a tolerance window of three AAs (Fig. 2a and b and Supplementary Fig. S6). While these results are still only modest in terms of absolute performance, it presents a significant improvement over PeptideLocator and can show promising accuracy on well-understood peptides (Fig. 2c–e).

When reporting performance separately for peptides and propeptides, we observe that DeepPeptide performs worse for propeptides than for peptides at tolerance windows of 0 and 1 (Supplementary Fig. S7). However, at higher tolerances the performance is comparable. This could be due to the reported start and end positions in UniProt not perfectly matching the actual likely site of cleavage, which is a common problem in peptide data and might be more pronounced in propeptides, where annotations are less scrutinized due to their perceived lower physiological relevance.

We evaluate both ESM-1b and the more recent ESM-2 (650 million) as pLMs. Additionally, we train the model on one-hot encoded AAs as a baseline. For this, we tokenize AA sequences using the tokenizer of the ESM model and one-hot encode each token as a vector of size 33. To save resources, these model ablation experiments were done in standard cross-validation using partition 0 as the test set. We observe that using last-layer representations of ESM-2 performs best on our task, as opposed to ESM-1b which benefited from using the penultimate layer instead (Table 2 and Supplementary Fig. S8). Due to their comparable performance, we trained both ESM-1b (L. 32) and ESM-2 (L. 33) in full nested cross-validation. This detailed analysis revealed that over the full dataset, the superior performance of ESM-2 over ESM-1b is mainly driven by an improvement in predicting propeptides (Supplementary Fig. S9).

**Table 2.** Performance of different sequence embedding methods with the DeepPeptide model.^a^

	Peptide borders ± 3		Propeptide borders ± 3	
Model	Precision	Recall	Precision	Recall
ESM-1b (layer 33)	0.62	0.39	0.59	0.42
ESM-1b (layer 32)	0.67	0.40	0.62	0.45
ESM-2 (layer 33)	**0.69**	**0.43**	**0.64**	**0.46**
ESM-2 (layer 32)	0.63	0.42	0.57	**0.46**
One-hot	0.50	0.18	0.47	0.25

a Performances were computed on split 0 of the nested CV scheme. The highest values are shown in bold.

To validate the performance of DeepPeptide in its likely use case, the discovery of peptides in proteomes without prior knowledge, we applied it to 48 curated reference proteomes of eukaryotic organisms from the Quest for Orthologs project (Altenhoff 2016). Throughout all organisms, we observe that the number of predicted peptides and propeptides is mostly higher than what is annotated in UniProt for the same set of proteins (Table 3 and Supplementary Table S3). As evident from the large number of proteomes that have zero peptides annotated, this is likely due the fact that peptides are vastly underannotated in UniProt, as their only source is manual curation from literature. Given the cross-validated precision of 0.69 of DeepPeptide, it can be assumed that the majority of the predicted peptides are correct, and the model reasonably estimates the order of magnitude of peptide prevalence in underannotated proteomes.

Notably, the results suggest that even well-researched model organisms such as *Danio rerio*, *Zea mays*, and *Oryza sativa* suffer from severe underannotation in UniProt and may benefit from peptide predictions.

## 4 Conclusion

We introduce DeepPeptide, a model for predicting potential endogenous peptides in mature protein sequences. DeepPeptide improves performance over previous approaches and allows for easier investigation of results when applied to large datasets, as it predicts discrete peptides with defined start and end positions, as opposed to a per-residue peptide probability score.

A remaining limitation of the model is that it cannot handle overlapping peptides explicitly. During training, we opted to randomly sample peptides from overlapping regions to avoid introducing any bias by data selection, but for inference the sequence-to-sequence framework of the CRF limits us to predicting one label at each position. The existence of overlapping peptides also presents a challenge for computing precision and recall, requiring us to simplify the objective to just measure whether any peptide in an overlapping group was predicted, rather than properly evaluating prediction of all existing peptides. Moreover, it is known that peptide cleavage in higher organisms is highly tissue specific, whereas DeepPeptide was trained on all known peptides and has no means of handling such specificities. Similarly, while pLM embeddings encode information about a sequence’s taxonomic origin, DeepPeptide does not explicitly take species information into account and might potentially predict peptides cleaved by processes that are not present in a given organism.

While showing a substantial improvement in performance over previous approaches, performance in absolute terms is still modest compared to related tasks such as signal peptide prediction. It can be assumed that peptide cleavage prediction remains a challenging prediction problem given the available UniProt annotations and would benefit from more comprehensive data. Of particular interest would be data where cleavage sites are resolved by proteases, as opposed to just treating all peptides and propeptides the same. This would enable more detailed modeling of substrate specificities and cleavage motifs and mitigate the risk of not being able to learn underrepresented cleavage motifs.

Although it was only trained on peptide-containing proteins, DeepPeptide predicts a credible number of peptides and propeptides when applied to whole proteomes. This indicates that the model correctly learned to focus on the characteristic features of peptides and does not suffer from an out-of-distribution performance gap when applied to proteins that are not peptide precursors. Thus, DeepPeptide can aid in the annotation of proteomes without prior knowledge about encoded peptides. DeepPeptide is available at ku.biolib.com/deeppeptide.

## Supplementary Material

btad616_Supplementary_Data

## Data Availability

The training dataset, codebase, and model weights are available at github.com/fteufel/DeepPeptide and https://zenodo.org/record/8352932.
