# Supplementary material for: DeepPeptide predicts cleaved peptides in proteins using conditional random fields
Source: Bioinformatics. 2023 Oct 9;39(10):btad616. doi: 10.1093/bioinformatics/btad616 (PMC10585352; doi:10.1093/bioinformatics/btad616)
Supplement: btad616_Supplementary_Data [file btad616_supplementary_data.zip › Supplement.docx]

Supplementary material for

DeepPeptide predicts peptide cleavage in proteins using conditional random fields

Felix Teufel, Jan Christian Refsgaard, Christian Toft Madsen, Carsten Stahlhut, Mads Grønborg, Ole Winther and Dennis Madsen


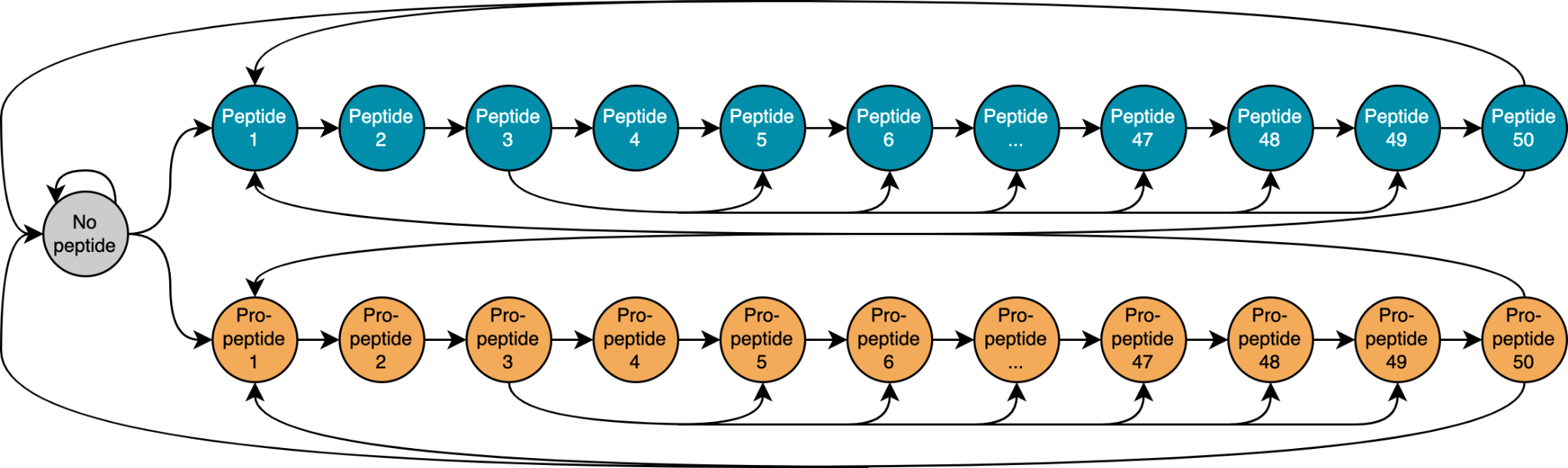


Figure S1. The state space model of the conditional random field.


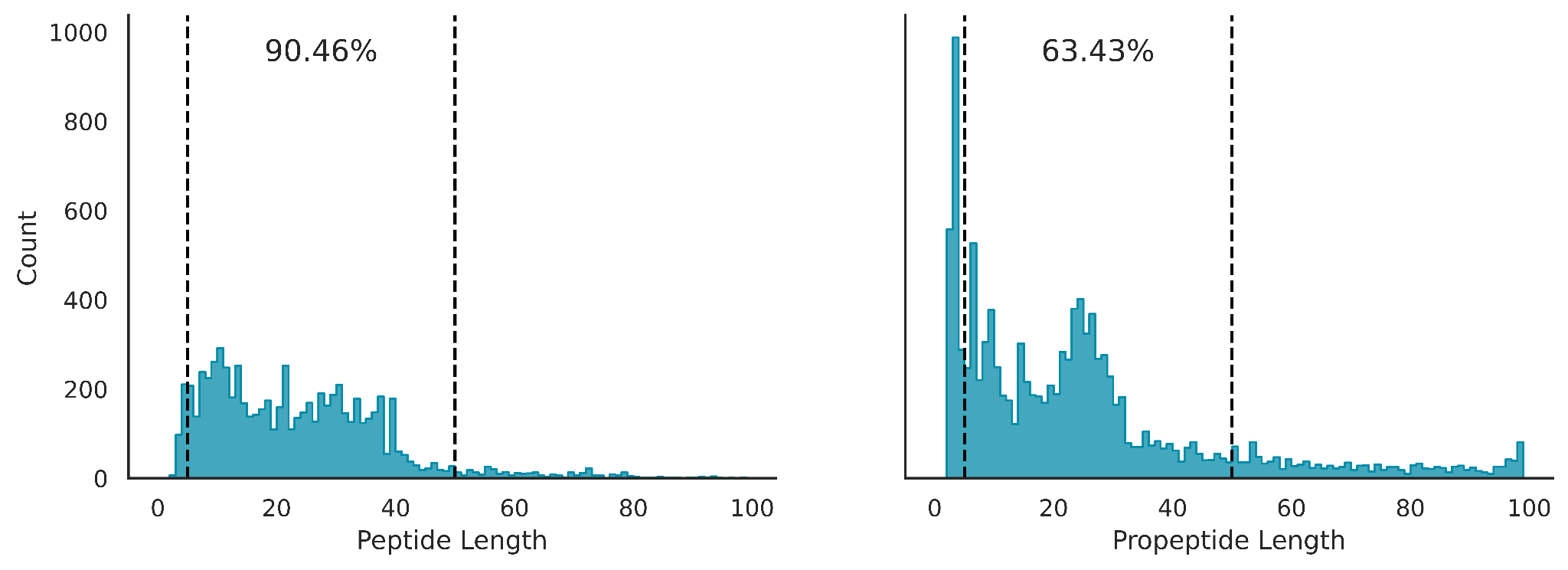


Figure S2. The length distributions of peptides and propeptides annotated in Uniprot that pass the data selection criteria.


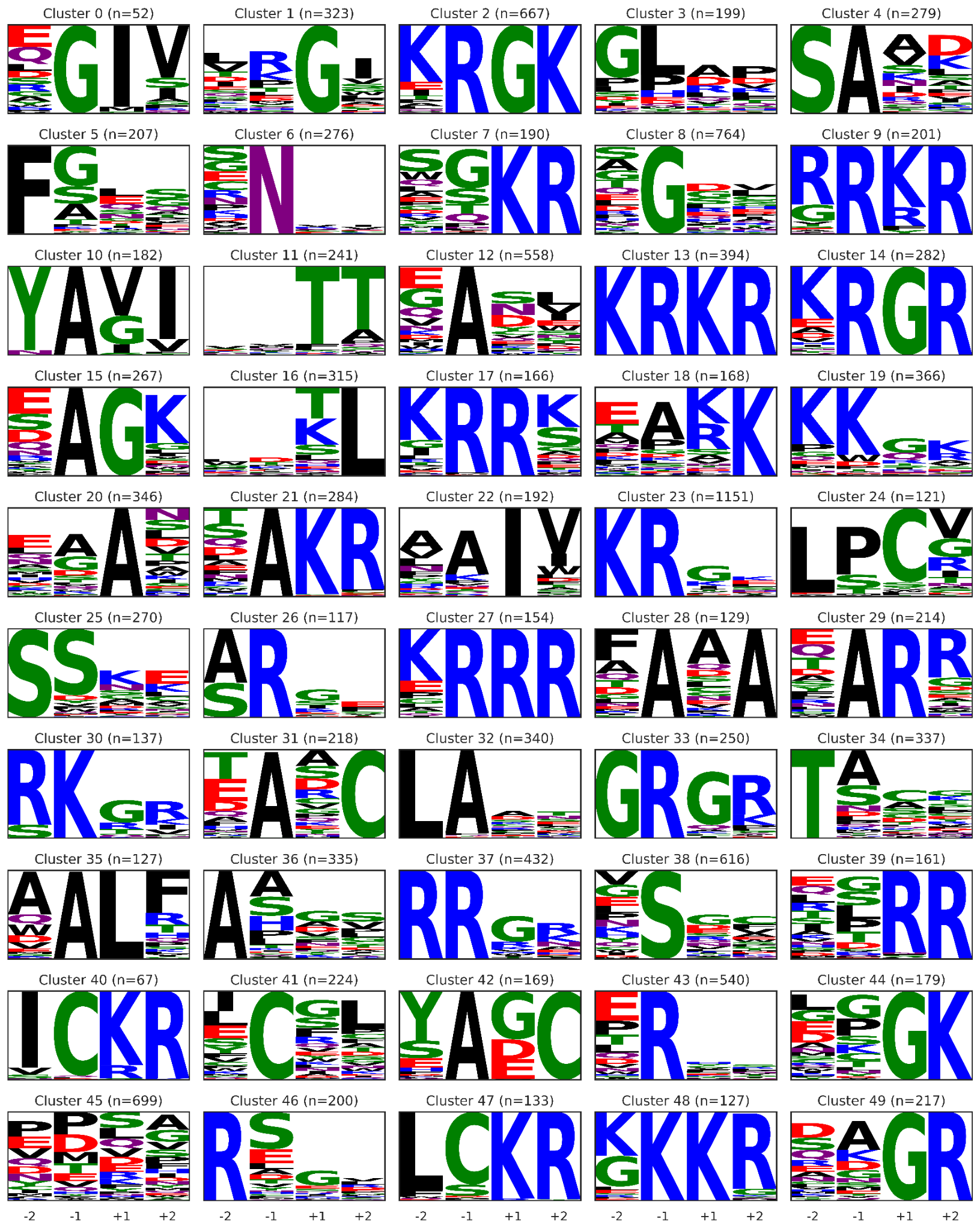


Figure S3. The clusters used for balancing peptides and propeptides by cleavage motifs between folds. -2 and -1 are the two AAs adjacent to the peptide’s N terminus, +1 and +2 are adjacent to the C terminus. The number of peptides and propeptides in each cluster is indicated.


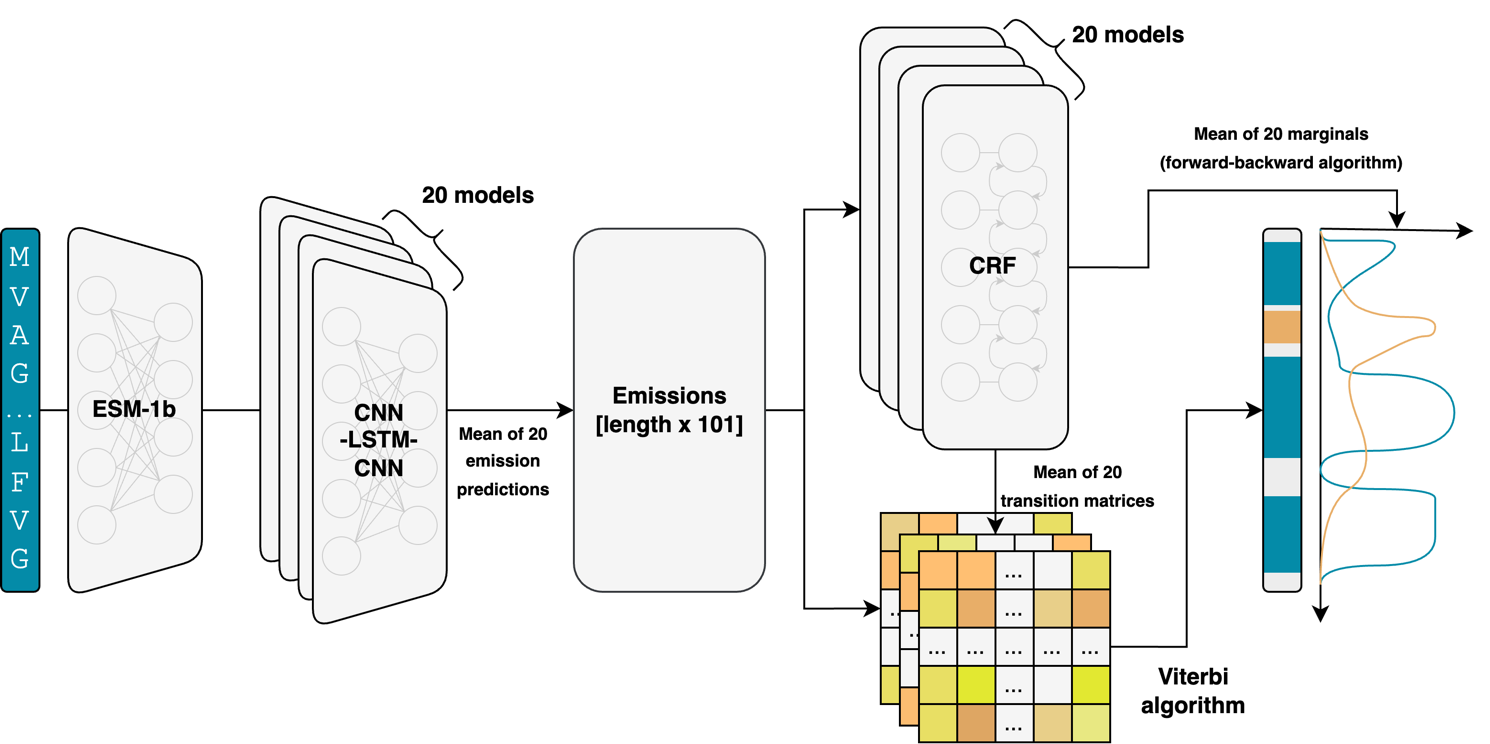


Figure S4. The ensemble strategy used to combine multiple CRF models obtained from nested cross-validation.


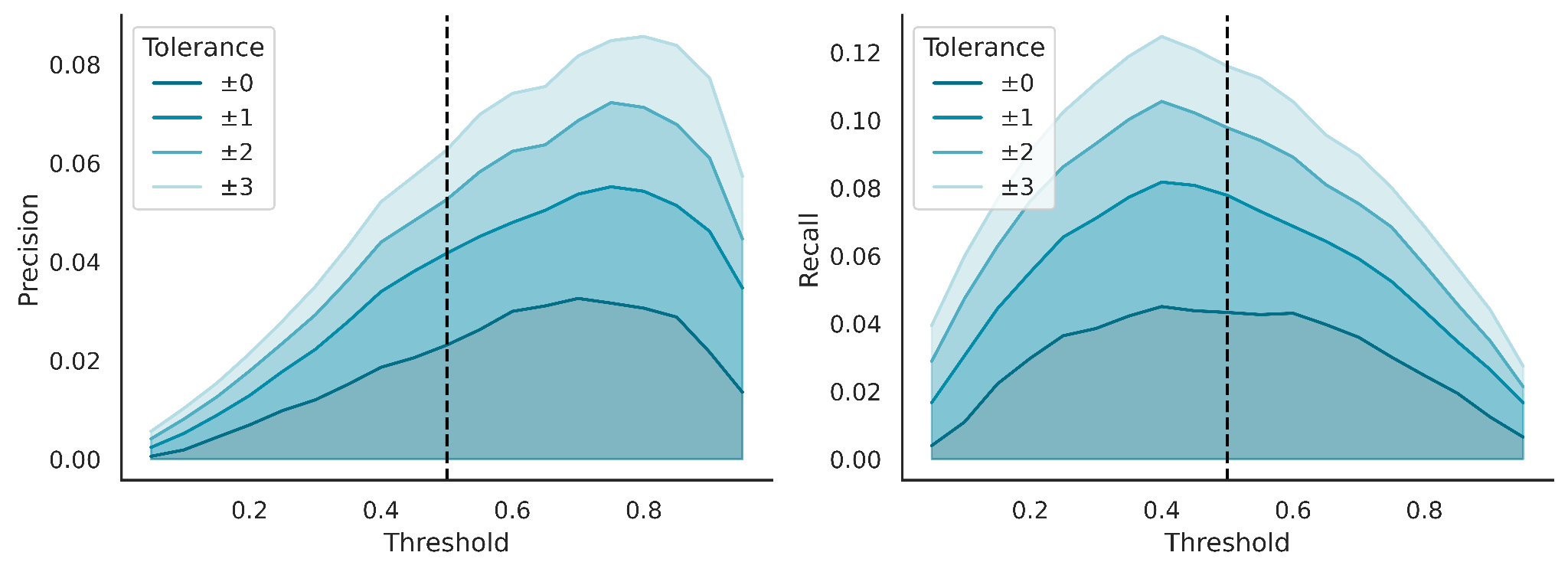


Figure S5. The effect of different prediction thresholds on PeptideLocator performance. The default threshold of 0.5 favors higher recall at the expense of lower precision.


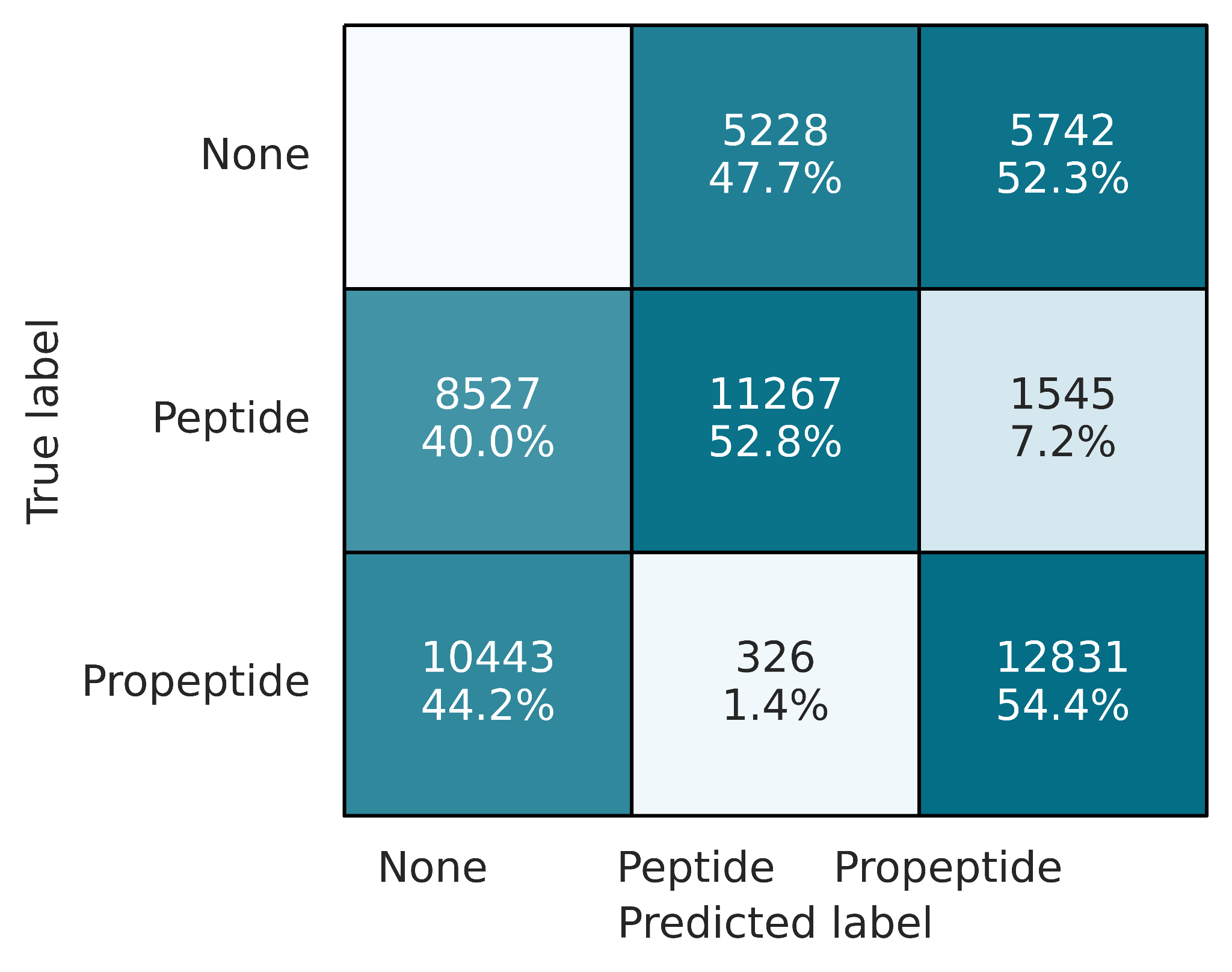


Figure S6. Confusion matrix of cross-validated DeepPeptide predictions at a tolerance threshold of 3.


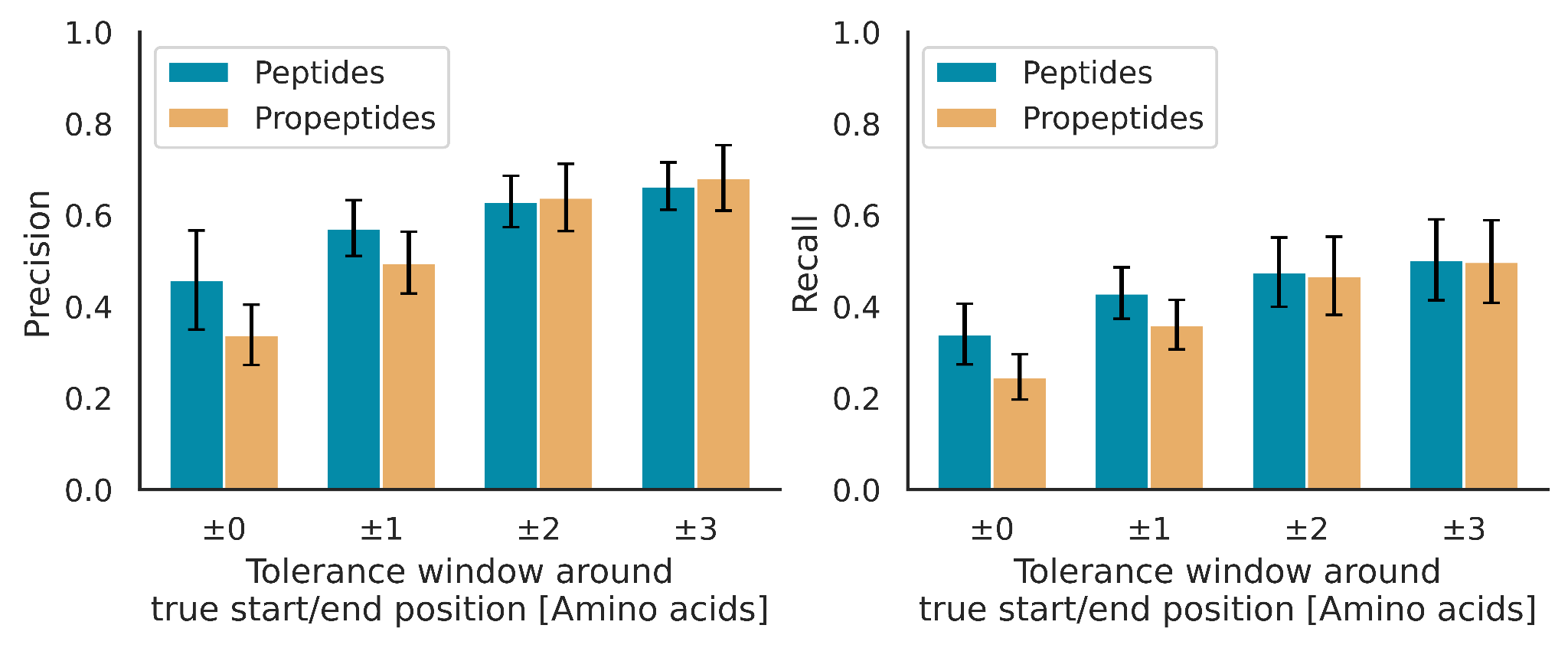


Figure S7. Performance of DeepPeptide computed separately for peptides and propeptides.


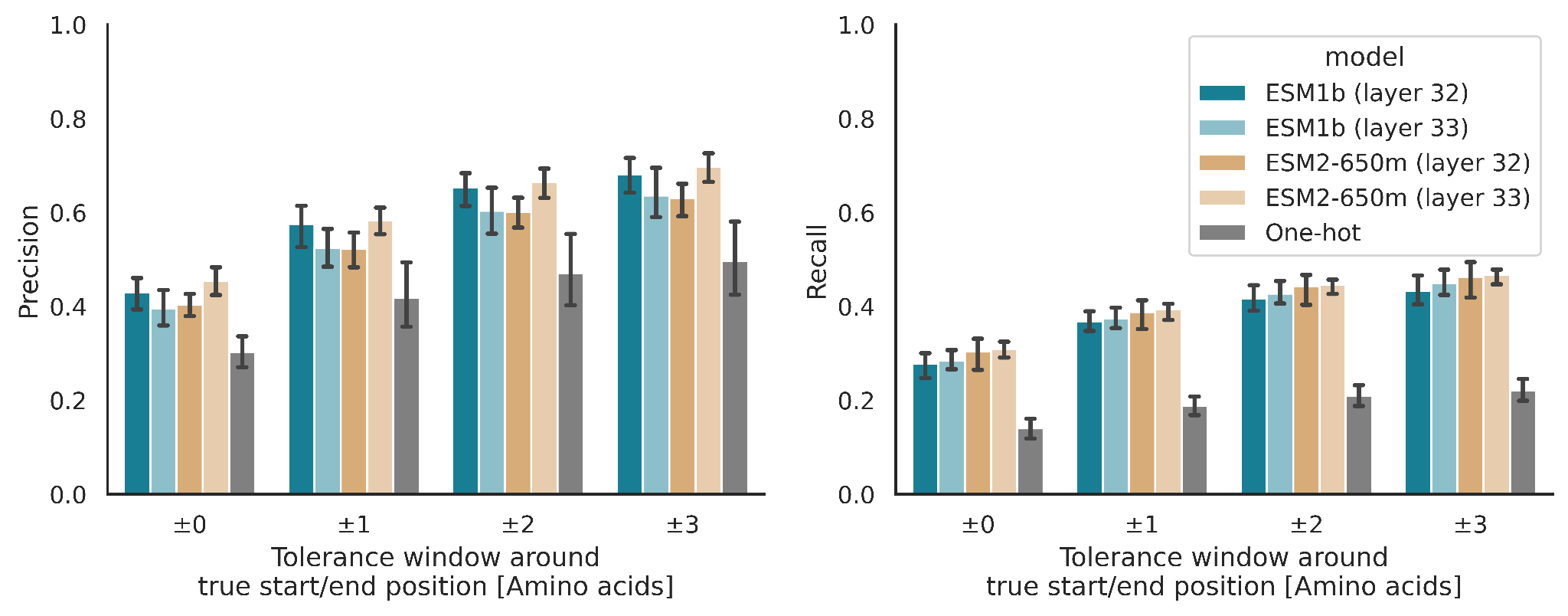


Figure S8. Performance of the DeepPeptide architecture when using ESM-1b and ESM-2 (650M) as LMs. Performance was measured using cross-validation with partition 0 as the test set.


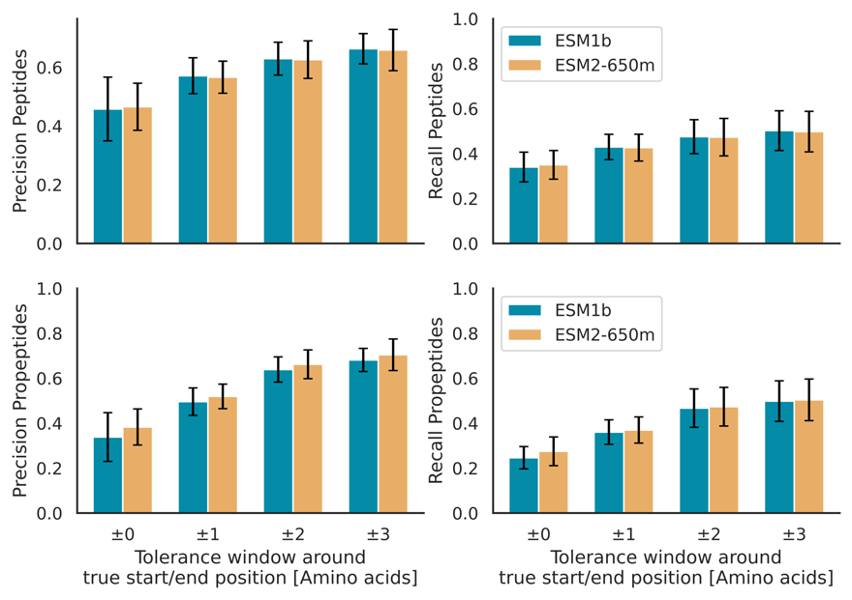


Figure S9. Full nested cross-validated performance of ESM-1b (layer 32) and ESM-2 (650M, layer 33)

Table S1. The hyperparameter space used for both the ESM-1b and ESM-2 (650M) based models. The space was explored in nested cross-validation (best set determined by average validation performance on 4 inner fold models).

| **Parameter** | **Lower bound** | **Upper bound** | **Distribution** |
| --- | --- | --- | --- |
| Learning rate | 0.0001 | 0.01 | log-uniform |
| Batch size | 10 | 100 | Step size 10 |
| Embedding dropout | 0 | 0.7 | Uniform |
| Convolution dropout | 0 | 0.7 | Uniform |
| Kernel size | 1 | 5 | Step size 2 |
| CNN channels | 40 | 128 | Step size 8 |
| LSTM hidden size | 16 | 192 | Step size 16 |

Table S2. Hyperparameters found by the hyperparameter search in nested cross-validation. Learning rates and dropouts were rounded.

| **Model** | **Learning rate** | **Batch size** | **Embedding dropout** | **Convolution dropout** | **Kernel size** | **CNN channels** | **LSTM hidden size** |
| --- | --- | --- | --- | --- | --- | --- | --- |
| T0 | 0.0033 | 90 | 0.2349 | 0.1041 | 5 | 96 | 48 |
| T1 | 0.0003 | 70 | 0.3255 | 0.3928 | 5 | 80 | 32 |
| T2 | 0.0010 | 50 | 0.1437 | 0.4085 | 3 | 80 | 32 |
| T3 | 0.0033 | 60 | 0.4781 | 0.5082 | 5 | 96 | 32 |
| T4 | 0.0055 | 20 | 0.6902 | 0.2672 | 5 | 48 | 48 |

**Supplementary Note S1: Decoding the Conditional Random Field**

A linear-chain conditional random field (CRF) models the conditional distribution p(Y|X), with Y being a sequence of labels, and X being a sequence of vector-valued inputs. Both sequences are of equal length T. For inference, dynamic programming algorithms can be used that yield exact solutions. Commonly, we are interested in finding the most likely path (more precisely: the maximum a posteriori estimate) of labels Y given an input X. This most likely path can be inferred using the Viterbi algorithm (Viterbi, 1967). Additionally, for interpretation of results, it can be helpful to also investigate the posterior marginal distribution of p(Y_t_|X) at each position t in the sequence. This distribution can be computed jointly for all t ∈ T using the forward-backward algorithm (Bishop, 1967).

The two algorithms differ from one another in the sense that Viterbi predicts the most likely sequence of states, without any position-wise probabilities, whereas forward-backward predicts the most likely label (and the associated probability distribution) at each position separately.

**References**

Viterbi, A. (1967) ‘Error bounds for convolutional codes and an asymptotical-ly optimum decoding algorithm’, IEEE Transactions on Information Theory, 13(2), pp. 260–269.

Bishop, C.M. and Nasrabadi, N.M. (2006) ‘Pattern recognition and machine learning’ Vol. 4, No. 4. New York: Springer.
